# Exploring the Antifungal Potential of Lawsone-Loaded Mesoporous Silica Nanoparticles Against *Candida albicans* and *Candida glabrata*: Growth Inhibition and Biofilm Disruption

**DOI:** 10.3390/jof11060427

**Published:** 2025-06-01

**Authors:** Fatemeh Nikoomanesh, Mahsa Sedighi, Mahdi Mahmmoodi Bourang, Mitra Rafiee, André Luis Souza dos Santos, Maryam Roudbary

**Affiliations:** 1 Infectious Disease Research Center, Birjand University of Medical Sciences, Birjand 9717853577, Iran; fateme.nikoomanesh@bums.ac.ir; 2 Medical Nanotechnology Department, Breast Cancer Research Center, Motamed Cancer Institute, ACECR, Tehran 1517964311, Iran; m.sedighi67@yahoo.com; 3 Student Research Committee, Birjand University of Medical University, Birjand 9717853076, Iran; mahdi_mahmoodi99@yahoo.com; 4 Department of Immunology, School of Medicine, Cellular and Molecular Research Center, Birjand University of Medical Sciences, Birjand 9717853076, Iran; rafiee64mitra@gmail.com; 5 Laboratório de Estudos Avançados de Microrganismos Emergentes e Resistentes, Departamento de Microbiologia Geral, Instituto de Microbiologia Paulo de Góes, Universidade Federal do Rio de Janeiro, Rio de Janeiro 21941901, RJ, Brazil; andre@micro.ufrj.br; 6 Rede Micologia RJ—FAPERJ, Instituto de Microbiologia Paulo de Góes, Universidade Federal do Rio de Janeiro, Rio de Janeiro 21941901, RJ, Brazil; 7 Sydney Infectious Diseases Institute, Faculty of Medicine and Health, University of Sydney, Sydney, NSW 2006, Australia; 8 Westmead Hospital, NSW Health, Sydney, NSW 2145, Australia

**Keywords:** antifungal activity, biofilm formation, *Candida albicans*, *Candida glabrata*, lawsone, mesoporous silica nanoparticles

## Abstract

The incidence of fungal infections is significantly rising, posing a challenge due to the limited class of antifungal drugs. There is a necessity to combat emerging resistant fungal infections by developing novel antifungal agents. This study aimed to evaluate the antifungal effects of lawsone (LAW), a natural component extracted from herbal medicine, and LAW-loaded mesoporous silica nanoparticles (LAW-MSNs) on growth, biofilm formation, and expression of *ALS1* and *EPA1* genes contributing to cell adhesion of *Candida* spp. Twenty *C. albicans* and twenty *C. glabrata* isolates, including ten fluconazole-resistant and ten fluconazole-susceptible isolates, were examined. The findings of the study indicated that LAW and LAW-MSNs inhibited *Candida* isolates growth at MIC range of 0.31–>5 µg/mL and significantly reduced biofilm formation in *C. albicans* and *C. glabrata*. Moreover, both LAW and LAW-MSNs downregulated the expression of the adhesion genes *ALS1* and *EPA1* in *C. albicans* and *C. glabrata.* Based on the obtained findings, LAW emerged as a promising antifungal candidate. However, the nano-formulation (LAW-MSNs) improved its antifungal properties.

## 1. Introduction

*Candida albicans* and *Candida glabrata* (now known as *Nakaseomyces glabratus*) are the two most prevalent etiologic agents of candidiasis worldwide [1,2,3]. Invasive candidiasis (IC) is associated with high mortality and morbidity in immunocompromised patients [3], particularly when co-infection of *C. albicans* and *C. glabrata* occurs [1,4,5]. In addition, emerging resistant species to first-line antifungal drugs (such as echinocandins and azoles) are leading to therapeutic failure and poor patient outcomes [6]. This issue is exacerbated by a limited number of effective antifungal drugs, drug–drug interactions, and drug toxicity [7,8]. Importantly, in 2022, the WHO listed *C. albicans* and *C. glabrata* as critical and high-priority pathogens on the fungal pathogen list (FPPL) to address the growing issue of antifungal resistance worldwide [9]. Noticeably, the ability for biofilm formation, a microbial consortium formed by *Candida* species adhering to both biotic and abiotic surfaces with extracellular polymeric substance, serves as a resistant structure that hinders the penetration of antifungal drugs, leading to persistent infections and treatment challenges [10]. Adhesion to the surfaces is the primary and most crucial stage in the process of *Candida* colonization and infection [11,12]. In *C. albicans*, the glycosylphosphatidylinositol (GPI)-anchored hypha-associated agglutinin-like sequence (Als) proteins interact with mammalian cadherins. In contrast, the epithelial adhesion (EPA) subfamily encodes major groups of adhesins in *C. glabrata* [11], which is structurally similar to Als proteins of *C. albicans* [13,14]. Given the increasing prevalence of drug-resistant isolates, biofilm formation ability, and the limited effectiveness of current antifungals, there is an urgent need to identify alternative antifungal agents that are more effective and tolerant than existing antifungals [15,16,17,18,19]. Designing original drugs from traditional plant-based medicines holds new promise for the future. Medicinal plants contain a variety of natural compounds with diverse biological activities, including antibacterial, antifungal, antiviral, anti-inflammatory, and antioxidant effects. Moreover, these plants generally have fewer side effects compared to synthetic drugs. Notably, there are no significant reports of potent toxins derived from commonly used medicinal plants and their compounds [20]. Iran, one of the richest countries in terms of biodiversity, boasts approximately 8000 medicinal plant species, reflecting its long history of traditional medicine use. Interestingly, Henna (*Lawsonia inermis*) has been traditionally used to treat infections caused by filamentous fungi, such as dermatophytosis, in Africa, Asia, the Middle East, Pakistan, India, and particularly in Iran [21]. Henna has active compounds that include beta-sitosterol glucoside, flavonoid, quinoid, naphthalene derivatives, xanthos, and phenolic glycoside. This plant is 25–33% soluble in water, and the plant contains an effective substance called Lawsone (LAW) [22]. Studies have shown that the ethanol extract of the whole plant exhibits both antifungal and antitubercular activities [23]. In general, research on the antimicrobial properties of LAW has primarily focused on leaf extracts, typically prepared using solvents such as methanol, ethanol, and chloroform [24]. The bioavailability, solubility, and effectiveness of LAW notably improved using nano systems [23]. Recent advancements in nanoparticle-based antifungal agents, emphasizing their potential application to revolutionize therapy, especially in combating *Candida* infections, pave the way for targeted treatment to improve clinical outcomes [25,26,27]. In response to the global call to fight fungal infections, the present study aimed to combat drug-resistant *C. albicans* and *C. glabrata* by synthesizing LAW-loaded mesoporous silica nanoparticles (LAW-MSNs) and assessing their effectiveness on growth, biofilm formation, and expression of *ALS1* and *EPA1* genes in fluconazole-susceptible and fluconazole-resistant clinical strains of *C. albicans* and *C. glabrata.*

## 2. Materials and Methods

### 2.1. Candida spp. and Culture Conditions

In total, forty clinical isolates, including twenty *C. albicans* (ten fluconazole-resistant and ten fluconazole-susceptible strains) as well as twenty *C. glabrata* (ten fluconazole- susceptible and ten fluconazole-resistant strains) were examined. We used *Candida* isolates from our previous studies, which were part of a fungal culture collection approved under an ethical code number: ir.bums.REC.1397.367. All isolates had been previously identified by PCR and were revived on Sabouraud Dextrose Agar (SDA) for further testing [28]. In addition, two standard strains of *Candida albicans* (ATCC10231) and *Candida glabrata* (ATCC2001) were used in all experiments. All isolates were sub-cultured on Sabouraud Dextrose Agar (SDA, Sigma-Aldrich, St. Louis, MO, USA) and incubated at 37 °C for 48 h to optimize the growth.

### 2.2. Preparation of Lawsone-Loaded Mesoporous Silica Nanoparticles (LAW-MSN)

In this study, we used a modified synthesis method to prepare MSNs [29]. First, the synthesis solution contained 360 mL of dH_2_O, 60 mL of ethylene glycol (DNA biotech, Tehran, Iran), and 10.9 mL of 30% ammonia, as well as 2.358 g of Cetyltrimethylammonium bromide (CTAB, Sigma-Aldrich, St. Louis, MO, USA). After that, 2.86 mL of tetraethylorthosilicate (TEOS) was added gently to the solution. The synthesis solution was stirred at 250 rpm for 2 h at 50 °C, and then it was kept at 50 °C overnight without stirring. The resulting particles were collected by centrifugation at 10,000 rpm for 15 min, washed at least three times with 100 mL of CTAB extraction solution (2 g of ammonium nitrate in 100 mL of ethanol), and sonicated in an ultrasonic bath (Powersonic S10, Bolton, ON, Canada) for 30 min. To load Lawsone (Sigma-Aldrich, St. Louis, MO, USA), MSNs were mixed by LAW solution (20 mL, 4 mg/mL). After stirring for 3 h at 300 rpm, the solution was centrifuged at 10,000 rpm for 20 min, washed three times with dH_2_O, and kept at room temperature until experiments.

### 2.3. Physicochemical Characterization of MSNs

The diameter of nanoparticles (NPs) was determined by transmission electron microscopy (TEM) (Philips, Eindhoven, The Netherlands). The porosity of MSNs was also investigated by the assessment of TEM images before and after CTAB removal. The crystalline structure of the samples was investigated using X-ray diffraction (XRD) (Smart LAB, Rigaku, Japan) analysis at 2θ of 10–80°, and small-angle X-ray scattering (SAXS) analysis was also performed at angles smaller than 10° [29].

### 2.4. Antifungal Susceptibility Testing (AFST) of LAW and LAW-MSN

Antifungal activity of LAW and LAW-MSNs was determined according to the Clinical and Laboratory Standards Institute (CLSI M27-A3/S4 [30,31]) guideline against both clinical isolates and standard strains [32]. For this, the serial concentrations of LAW and LAW-MSNs (10–0.01 µg/mL) were prepared in RPMI1640 medium, and 100 µL was added to 96-well polystyrene microtiter plates. Cell suspension was adjusted to 0.5 × 10^6^–2.5 × 10^6^ cells mL^−1^ and 100 µL added to each well. A well containing RPMI and normal saline (NS) is considered a negative control, whereas a well containing RPMI with yeast suspension is a positive control. Microplates were incubated at 37 °C for 24 h. At the end of this incubation, the minimum inhibitory concentration (MIC50) was determined visually as the inhibition of 50% yeast growth compared to the positive control. Fungicidal effect of LAW and LAW-MSNs was determined by colony count on SDA. All experiments were performed in triplicate.

### 2.5. Effect of LAW and LAW-MSN on Biofilm Formation

The effect of LAW and LAW-MSNs on biofilm formation of *C. albicans* and *C. glabrata* was evaluated using colorimetric the MTT (Tetrazolium salt 3-[4, 5-dimethylthiazolyl-2]-2, 5-diphenyltetrazolium bromide) (DNA biotech, Tehran, Iran) assay, previously described by Nikoomanesh et al. [12]. In brief, for biofilm formation, 10 µL (10^3^ cells/mL) of yeast cells, both fluconazole-resistant and susceptible *C. albicans* and *C. glabrata* isolates, were inoculated in a 96-well U-bottom microplate, containing 200 µL of RPMI1640 medium treated with the obtained MIC50 concentrations of LAW and LAW-MSNs. The plates were incubated at 37 °C for 1.5 h at 75 rpm, allowing the cells to attach to the wells. Following the attachment phase, unattached cells were removed by washing the wells with 150 uL of phosphate-buffered saline (PBS), and 100 μL of fresh RPMI 1640 medium (DNA biotech, Tehran, Iran) was added to each well. The plate was incubated at 37 °C for 72 h and shook at 75 rpm to allow for the growth of the biofilms. After biofilm formation, the wells were washed twice with 200 μL PBS. Then, 200 μL of PBS and 12 μL of the MTT were added to each well and incubated again at 37 °C for 4 h. After this period, biofilm was determined by measuring optical density (OD) at 570 nm by using an ELISA reader (Bio-Rad, California, USA). Wells containing RPMI with yeast suspension (without compound treatment) were positive controls, whereas wells with RPMI without suspension were considered negative controls during tests. Biofilm formation percentage was determined using the following formula:

N1/N2 × 100, (N1 is the mean LAW and LAW-MSNs -treated isolates, and N2 is the mean LAW and LAW-MSNs -untreated isolates). Finally, the ability of biofilm formation in fluconazole-susceptible and resistant strains was compared to the control group.

### 2.6. Quantitative Real-Time Polymerase Chain Reaction (qRT-PCR)

The expression of *ALS1* and *EPA1* genes in *C. albicans* and *C. glabrata*, respectively, was evaluated using qRT-PCR. The specific primers for *ALS1*, *EPA1,* and *Act1* as an internal reference gene were designed by using Allele ID primer design software (version 7.5, Table 1). In brief, fresh colonies of *C. albicans* and *C. glabrata* (10^3^ cells/mL) were exposed to MIC50 concentration of LAW and LAW-MSNs in a 96-well round bottom microtiter plate and incubated for 24 h at 37 °C. After that, yeast cells were harvested and washed with PBS and then the total RNA was extracted from yeasts with glass beads and lysis buffer, as described earlier [27]. Moreover, a cDNA template was synthesized using a cDNA synthesis kit (Parstoos, Mashhad, Iran), according to the manufacturer’s protocol. Quantitative real-time PCR was accomplished using AMPLIQON (Real Q plus 2 × master mixes Green High Rox) (Sinnagene, Tehran, Iran). To analyze PCR performance, the mixture containing 12.5 µL of master mix), 0.25 µL of each specific primer, and 4 µL of cDNA template (10 ng) was adjusted to a final volume of 25 µL using DEPC water. PCR conditions were 95 °C for 5 min, followed by 40 cycles of 95 °C for 15 s, 58 °C for 30 s, and 72 °C for 30 s using the ABI One Step machine (Applied Biosystems, Rotkreus, Switzerland). The expression levels of genes (*ALS1* and *EPA1*) before and after treatment were evaluated using the 2^−∆∆CT^ method, where Ct was the average threshold cycle number from three independent experiments. The threshold value > 1-fold was considered overexpression. Data were presented as the fold change in gene expression normalized to the *Act1* gene as the internal control.

### 2.7. Cell Viability Assay

LAW’s effects on the viability of the normal human fibroblast (HDF) cell line (Comprehensive Research Laboratory, Birjand University of Medical Sciences, Birjand, Iran) were assessed using the MTT assay. RPMI 1640 culture supplemented with 1% penicillin/streptomycin and 10% fetal bovine serum (FBS) (Sigma-Aldrich, St. Louis, MO, USA). In 96-well plates, 1 × 10^4^ cells were planted per 100 µL. Following 24 h, the cells were co-cultured with different doses of LAW (2–10 µg/mL) and incubated at 37 °C for 24 h with 5% CO_2_. After this time, 5 mg/mL of MTT (Sigma-Aldrich, St. Louis, MO, USA) working solution was added to each well, and the plate was incubated for 4 h. The formazan crystals were then dissolved with DMSO. A scanning spectrophotometer (Biotek Epoch, Winooski, VT, USA) was used to measure the absorbance at 570 nm [33].

### 2.8. Statistical Analysis

The collected data were statistically analyzed by SPSS (V. 22) software. Descriptive statistical tests and *t*-test were used to analyze the data. The normality of numeric variables was checked by the Kolmogorov–Smirnov test. The association between the antifungal activity of LAW and LAW-MSNs against *Candida* isolates was identified by the Chi-square test in univariate analysis. *p* < 0.05 was considered statistically significant.

## 3. Results

### 3.1. LAW-MSNs Synthesis and Characterization

**Figure 1:**
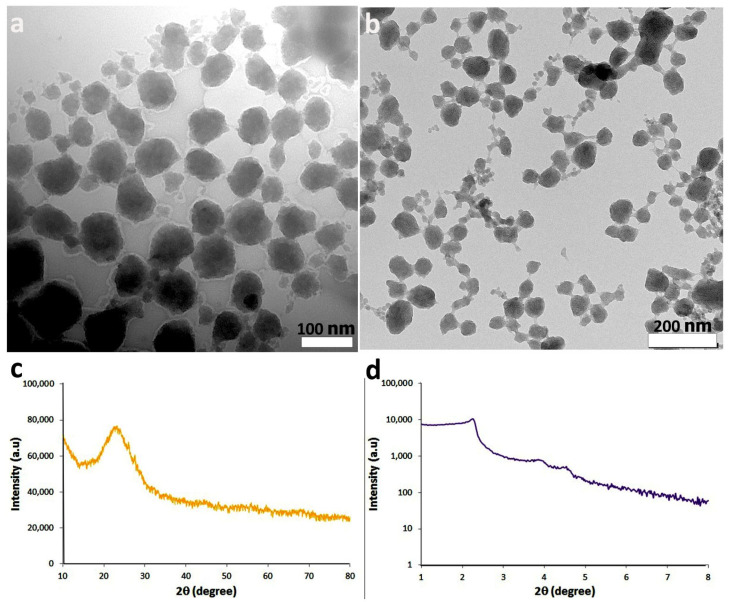
Characterization of synthesized MSNs by TEM: (**a**) before (scale bar = 100 nm) and (**b**) after (scale bar = 200 nm) CTAB removal, and XRD: (**c**) large angle XRD and (**d**) small angle XRD (SAXS).

Morphology and precise size of LAW-MSNs were investigated using TEM analysis. After the synthesis of MSNs, CTAB should be removed from the structure of MSNs to create the pores. Figure 1a shows the MSNs before removing CTAB, followed by the reaction. The pores are not visible due to the presence of the template molecule. After removing CTAB, the MSNs had a very uniform dispersion and a porous structure with nanometer pores, which are suitable for drug loading (Figure 1b). Crystalline structure was assessed by using XRD analysis with large and small angles. From the XRD pattern (Figure 1c), a peak is observed at 23°, which indicates the amorphous nature of NPs and confirms the absence of any impurity with NPs. The SAXS pattern (Figure 1d) of MSNs showed a strong diffraction peak at about 2.2° with Miller indices (100) and two other weaker peaks belonging to planes with Miller indices (110) and (200), which is characteristic of the hexagonal structure of pores.

### 3.2. Antifungal Activity of LAW and LAW-MSNs Against Candida Isolates

Minimum inhibitory concentration (MIC) and minimum fungicidal concentration (MFC) of LAW and LAW-MSNs against 20 *C. albicans* as well as 20 *C. glabrata* (each group 10 fluconazole-susceptible and 10 fluconazole-resistant strains) were evaluated by the CLSI M27/S4 protocol [31]. The results of MIC and MFC are represented in Table 2.

### 3.3. Effect of LAW and LAW-MSNs on Biofilm Formation

**Figure 2:**
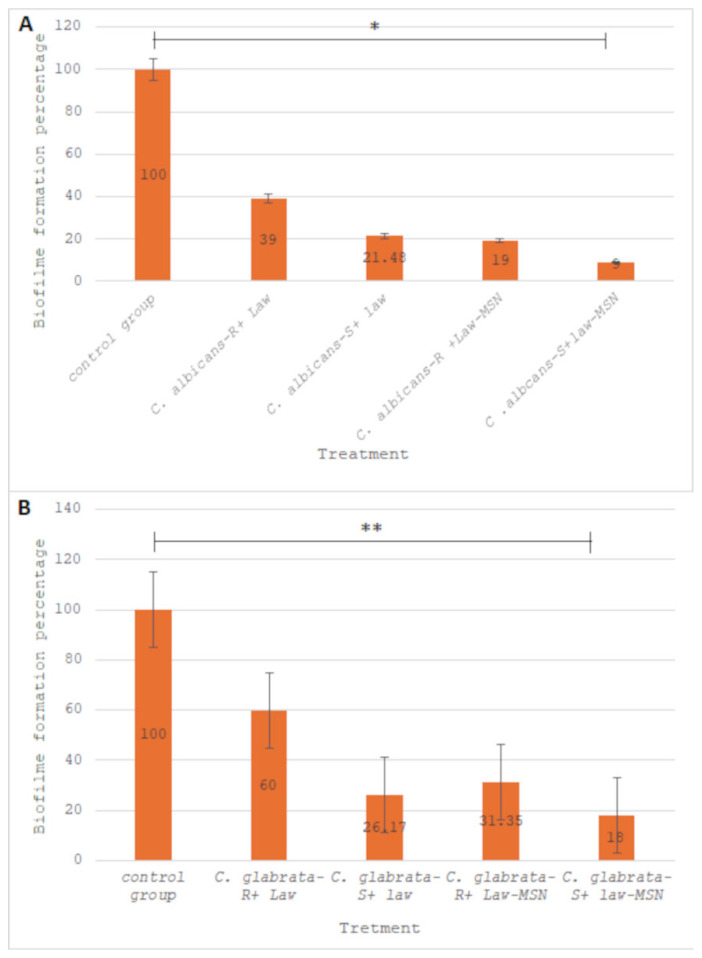
The percentage of biofilm formation in fluconazole-susceptible (*C. albicans*-S) and fluconazole-resistant (*C. albicans*-R) isolates (**A**), and fluconazole-susceptible (*C. glabrata*-S) and fluconazole-resistant (*C. glabrata*-R) isolates (**B**) treated with LAW and LAW-MSNs was compared to untreated cells (positive control, representing 100% biofilm formation capacity). Treatment with LAW and LAW-MSNs significantly reduced biofilm formation. Statistical significance was indicated as follows: * *p* < 0.001, ** *p* < 0.05 compared to the positive control (Student’s *t*-test).

Biofilm formation of fluconazole-susceptible and fluconazole-resistant strains of *C. albicans* and *C. glabrata* in the presence of LAW and LAW-MSNs at MIC50 concentration was determined (Figure 2A,B). The findings demonstrated that both LAW and LAW-MSNs exhibit anti-biofilm activity against resistant and susceptible *C. albicans* isolates at the tested MIC concentration (0.62 µg/mL and 1.25 µg/mL, respectively). Furthermore, LAW-MSNs significantly reduced biofilm formation to the lowest level of 9% in the fluconazole-susceptible *C. albicans* group compared to the control group (untreated cells) (*p*-value = 0.001, Figure 2A). Similarly, biofilm formation was significantly reduced in *C. glabrata* strains treated with LAW and LAW-MSNs at MIC of 1.25 µg/mL and 2.5 µg/mL, respectively. The most effective inhibition was observed in fluconazole-susceptible *C. glabrata* isolates treated with LAW-MSNs, where biofilm formation decreased to 18% compared to the control group (*p*-value = 0.035, Figure 2B).

### 3.4. ALS1 Gene Expression

**Figure 3:**
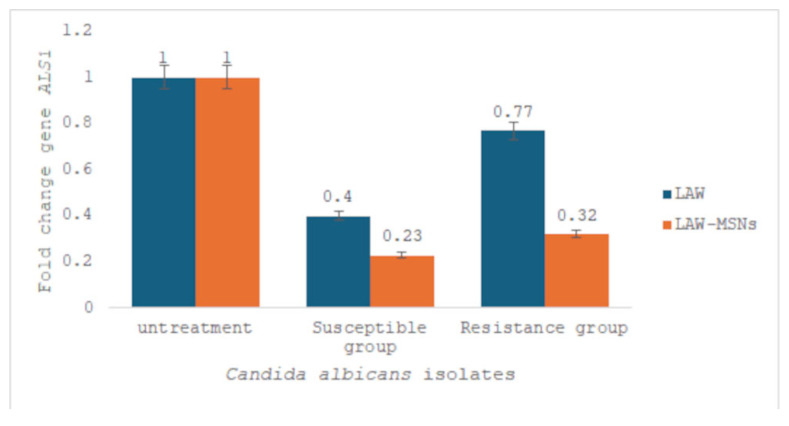
Comparison of *ALS1* gene expression in resistant and resistant-susceptible *C. albicans* isolates before and after treatment with the MIC of LAW and LAW-MSNs compared to untreated group related to *Act1* gene (housekeeping gene).

The gene expression levels in *Candida* isolates were evaluated using a real-time PCR assay. Data analysis revealed that the *ALS1* gene was downregulated in both fluconazole-susceptible and fluconazole-resistant *C. albicans* isolates treated with LAW and LAW-MSNs at the MIC (0.62 µg/mL and 1.25 µg/mL, respectively). Notably, cells treated with LAW-MSNs exhibited the highest efficacy in downregulating the *ALS1* gene, with fold changes of 0.2 and 0.3 in both treated fluconazole-susceptible and fluconazole-resistant *C. albicans*, respectively (Figure 3).

### 3.5. EPA1 Gene Expression

**Figure 4:**
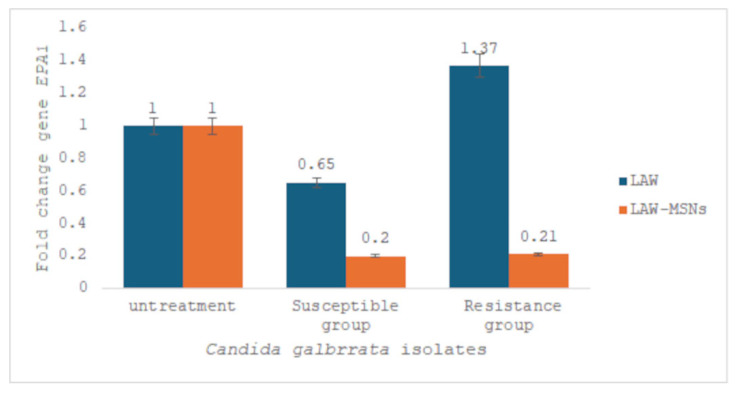
Comparison of *EPA1* gene expression in resistant and resistant-susceptible *C. glabrata* isolates before and after treatment with the MIC of LAW and LAW-MSNs compared to untreated group related to *Act1* gene (housekeeping gene).

Real-time PCR analysis revealed that the *EPA1* gene in fluconazole-susceptible and fluconazole-resistant *C. glabrata* isolates treated with LAW-MSNs at the MIC (1.25 µg/mL and 2.5 µg/mL, respectively) was downregulated compared to the untreated group (0.2-fold change). However, in fluconazole-resistant *C. glabrata*, treatment with LAW did not result in any downregulation of the *EPA1* gene expression compared to the control group (untreated strains) (Figure 4).

### 3.6. Viability of HDF Cells Exposed to LAW

**Figure 5:**
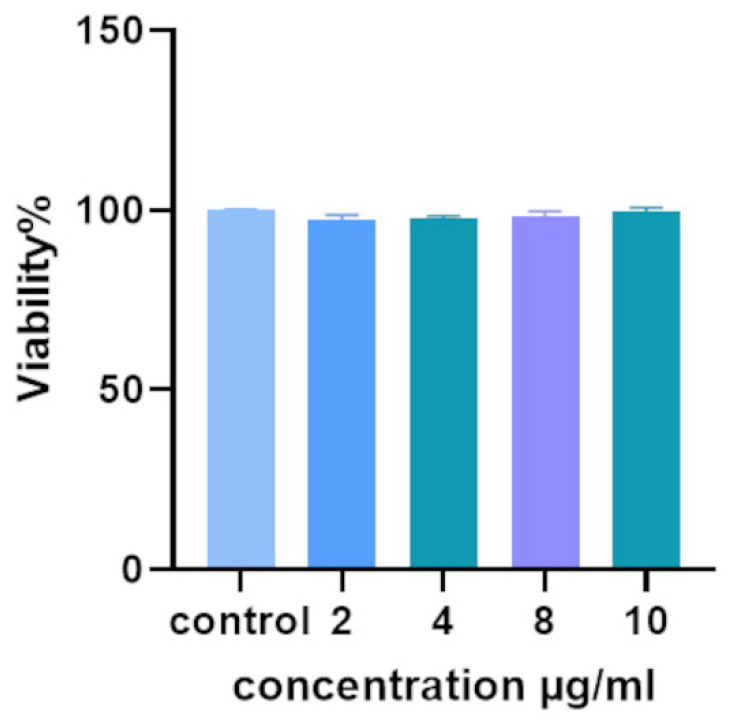
HDF cells were treated with various concentrations of LAW (2–10 µg/mL), and cell viability was assessed after 24 h. No cytotoxic effects were observed at tested concentrations compared to the untreated control group.

HDF cells were treated with varying concentrations of LAW and evaluated using the MTT assay. LAW at concentrations ranging from 2 to 10 µg/mL showed no significant differences (*p*-value > 0.05) in cytotoxic effects on normal human fibroblast cells compared to untreated cells (Figure 5).

## 4. Discussion

In recent decades, there has been a growing interest in herbal medicine as a desirable therapeutic option for infectious diseases, due to its limited side effects compared to existing synthetic drugs. Natural compounds play a crucial role in overcoming the challenge of antifungal resistance [17,18,34]. Additionally, drug delivery strategies involving nanoparticles and/or nano compounds have improved targeted therapy, enhanced antifungal activity, and reduced the cytotoxicity of natural-based compounds against drug-resistant infections. This is a significant concern in the healthcare system for efficient treatment [27,35,36]. Recent studies have investigated the effectiveness of hydroxynaphthoquinones (NQ), such as Lawsone (LAW), with various biological activities, including anti-inflammatory, antibacterial, and anticancer properties. While the antibacterial activity of LAW is well known, its antifungal activity has been reported in limited literature [20,22,33,37]. To address therapeutic approaches to drug-resistant fungal infections, we synthesized LAW-MSNs with the aim of enhancing the solubility and bioavailability of LAW. In our study, the nano-formulation (LAW-MSNs) demonstrated a marked inhibitory effect on *Candida* isolates, achieving lower minimum inhibitory concentrations (MICs) compared to LAW alone. Additionally, it significantly reduced biofilm formation in both fluconazole-resistant and fluconazole-susceptible isolates.

According to the literature, only limited data have reported the antifungal activity of LAW on some fungal species, whereas nano-capsulated LAW has not been investigated against *Candida* spp. to date. In agreement with our findings, some similar studies support our result. For instance, the study performed by Rahmoun et al. [23] showed LAW at 12 µg/mL and 50 µg/mL had an antifungal effect on *Fusarium graminarum* and *Aspergillus flavus*, respectively. Another report by Nawasrah et al. [38] showed Henna extract inhibited the biofilm structures of *Candida* stomatitis on acrylic resin, and at higher concentrations, the extract effectively inhibited the growth of *Candida*. Similarly, Yaralizadeh et al. [22] investigated the antifungal effect of LAW compared to clotrimazole as the control group in vivo, in which the treatment of rats with 2% extract of *Lawsonia inermis* over one week showed more effective therapeutic activity than clotrimazole treatment at doses of 2% and 4% in eliminating candidiasis vulvovaginitis. In addition, in a study by Singla et al. [39], Henna leaves extract had the maximum zone of inhibition (mean 20 mm). It has been shown that the antifungal activity of LAW is due to their chemical and medicinal properties by producing ROS as superoxide, hydroxyl radical, and hydrogen peroxide; ROS can trigger fungal cell death [40].

Given the advantages of nanotechnology strategies to increase drug solubility, bioavailability, and drug effectiveness, in this study, we synthesized MSNs, which are of great interest to drug delivery systems [41,42]. Barani et al. [37] demonstrated nano-formulation LAW-MSNs, overcoming the limitations of LAW, including low bioavailability, poor permeability, and instability in biological environments. Similarly, synthesized LAW-MSNs demonstrated both effective antifungal activity and biofilm inhibition compared to LAW, making it a potential candidate for future studies in vitro and in vivo against fungal species. There are notable differences between *C. albicans* and *C. glabrata* biofilms. *C. glabrata* biofilms consist solely of yeast cells, while mature *C. albicans* biofilms comprise a dense network of pseudohyphae, hyphae, and yeast cells. These differences in structure, cell morphology, extracellular polymeric substances (EPS), and overall dimensions affect the efficacy of antifungal compounds against the biofilms of these two species [43]. This is supported by the MIC results, which show that *C. glabrata* needs higher concentrations for growth inhibition compared to LAW and LAW-MSNs.

Although both LAW and LAW-MSNs exhibited antifungal activity and downregulated the expression of the adhesion genes *ALS1* and *EPA1* in *C. albicans* and *C. glabrata*, LAW-MSNs demonstrated significantly greater effectiveness. Our findings are in line with previous reports, like Rasoulian et al.’s study [21], which exhibited a stable formulation of solid lipid nanoparticles containing LAW that boosts the physical and chemical properties, such as antimicrobial agents, in in vivo experiments.

Finally, due to the limitations, this study only evaluated the viability of HDF cells exposed to LAW and found no cytotoxic effects at various concentrations tested. Further systematic studies will be conducted in the future to investigate the potential activity of LAW-MSNs. Indeed, evaluating the combination of LAW and LAW-MSNs with fluconazole may reveal potential synergistic effects, warranting further investigation in future studies.

## 5. Conclusions

Altogether, findings from this study revealed that LAW and LAW-MSNs remarkably inhibited the growth of both fluconazole-susceptible and fluconazole-resistant *C. albicans* and *C. glabrata* isolates. Nano-formulation of LAW-MSNs effectively inhibited biofilm structures of *Candida* isolates compared to LAW. Notably, *ALS1* and *EPA1* genes contribute to the adhesion of *Candida* isolates to host cells, down-regulated by exposure to LAW and LAW-MSNs. Consequently, nano-formulation of LAW-MSNs with enhanced antifungal properties could be a promising and predominant antifungal candidate with beneficial impact. More in vitro and in vivo studies are required to evaluate the effectiveness of LAW as a therapeutic option to address novel antifungal therapy in the future.

## Figures and Tables

**Table 1:** The primer sequences used in this study.

Gene	Primer 3′→5′
***ALS1***	Forward GAC TAG TGA ACC AAC AAA TAC CAG Reverse ACC AGA AGA AAC AGC AGG TG
***EPA1***	Forward TTC AGA CCA AAA GTA ACT GGC TTC Reverse CCT AAT AGG GTA ATA TAGC GCC CG
***ACT1***	Forward CCA GCT TTC TAC GTT TCC Reverse CTG TAA CCA CGT TCA GAC

**Table 2:** MIC and MFC (µg/mL) of LAW and LAW-MSNs against *Candida* isolates according to CLSI M27/S4 [31].

*Candida* Isolates	Compounds			
	LAW		LAW-MNS	
	MIC Range	MFC Range	MIC Range	MFC Range
Susceptible *C. albicans* isolates (n = 10)	0.31–1.25	0.62–2.5	0.31–0.62	0.62–1.25
Resistant *C. albicans* isolates (n = 10)	1.25–2.5	2.5–5	0.31–0.62	0.62–1.25
Susceptible *C. glabrata* isolates (n = 10)	0.62–2.5	1.25–5	0.31–1.25	0.62–2.5
Resistant *C. glabrata* isolates (n = 10)	2.5–5	2.5–5	1.25–2.5	2.5–5

## Data Availability

The original contributions presented in this study are included in the article. Further inquiries can be directed to the corresponding author(s).
